# Quality of pediatric anesthesia: A cross-sectional study of a university hospital in a low-income country

**DOI:** 10.1371/journal.pone.0194622

**Published:** 2018-04-09

**Authors:** Oskar Andersson, Peter Radell, Victor Ringo, Moses Mulungu, Tim Baker

**Affiliations:** 1 Department of Pediatric Peri-operative Medicine and Intensive Care, Karolinska University Hospital, Stockholm, Sweden; 2 Department of Physiology and Pharmacology, Section of Anesthesia and Intensive Care, Karolinska University Hospital, Stockholm, Sweden; 3 Emmilio Mzena Memorial Hospital, Dar es Salaam, Tanzania; 4 Muhimbili National Hospital, Dar es Salaam, Tanzania; 5 Department of Public Health Sciences, Global Health, Karolinska Institute, Stockholm, Sweden; 6 Department of Peri-operative Medicine & Intensive Care, Karolinska University Hospital, Stockholm, Sweden; University of Bern, University Hospital Bern, SWITZERLAND

## Abstract

**Objective:**

To evaluate the quality of pediatric anesthesia in a university hospital in Dar es Salaam, Tanzania.

**Method:**

A cross-sectional study conducted using a new tool that was developed from the literature and WHO recommendations including 28 parameters as standards for pediatric anesthesia. These 28 parameters consisted of 17 structure parameters of the equipment and medicines that should be present in theatre before any surgery starts, and 11 process parameters of actions taken by staff. Adverse events occurring during the anesthesia were recorded.

**Results:**

30 patients were included, aged between 1.5 months to 5 years with a mean of 2.4 years. 26 of the patients underwent elective surgery and 4 patients emergency surgery. Nine parameters were always present and one parameter (bag and mask) was not available for any of the patients. The structure index ranged from 71% to 94% with a mean of 84%. The process index had a mean score of 71% with a range from 50% to 90%: lower than the structure index (p<0.001). With the structure and process index combined the average score was 79% with a low of 67% and high of 89%. 70 adverse events were observed with a range from 0 to 7 adverse events per patient. The most common adverse event was hypoxia at extubation in 20 (69%) patients. Nine patients had an episode of severe hypoxia at extubation.

**Conclusion:**

Pediatric anesthesia in low resource settings suffers from deficiencies in the structures and processes of providing good quality care. Improvement efforts may be best focused on improving the consistency and quality of the process of care and a reduction in adverse events rather than the structures available. Use of the assessment tool developed for this research could be useful for systematic quality-improvement efforts and to assess the needs in different settings.

## Introduction

Pediatric anesthesia in low-income countries is under-resourced and can lack quality in low-income countries [1, 2]. Essential and safe surgery are receiving increased attention as surgical morbidity and mortality are recognized as comprising a significant share of the global burden of disease [3]. The lack of infrastructure and supplies can affect the ability to give safe surgical care: a review of eight low- and middle-income countries found that a functioning anaesthesia machine was available in only 33% of the facilities. [4]

Although improvements in some settings have lowered the risk of dying due to anesthetic complications, the risk of dying during anesthesia is 100–1000 times higher in some low-income countries compared with high-income countries. [3] One study from Benin in 2007 reported high morbidity (156 cardiac arrests per 10000 anaesthetics) and mortality (97 deaths per 10000 anaesthetics) during anaesthesia. [5] Hypoxia was the most commonly occurring problem leading to cardiac arrest and death. The use of monitoring was low and the anaesthesia was performed mainly by nurses working alone. A lack of appropriate drugs contributed to the high mortality and morbidity. [5]

In 2009 the WHO published Guidelines for Safe Surgery, outlining the need for trained anesthetists, equipment and monitoring. [3] There are, however, no standards for the structure or processes of good quality pediatric anesthesia in resource-limited settings. The goal of this study was to develop such standards and to assess the quality of the structure and processes of pediatric anesthesia in a hospital in Tanzania.

## Method

We conducted a cross-sectional study of pediatric anesthesia care at Muhimbili National Hospital (MNH) in Dar es Salaam, Tanzania. Tanzania is a low-income country in East Africa with 28.2% of the 51.8 million population living under the national poverty line [6] and an under-five mortality rate of 51.8 per 1000 live births [7]. MNH is the largest hospital in the country and functions as a national referral hospital. Data were collected during a 7-week period in February-April 2012 in the main surgery department which has six elective and one acute operating theatre.

Children under the age of 5 years who underwent surgery during the study period were included in the study. The children were categorized in groups for ear, nose and throat (ENT) or general pediatric surgery (PS). Study data were collected Monday to Friday, 9am to 5pm. One researcher (OA) was present in the operating theatres in order to observe care without interference and to fill in the data collection tool. If two children had surgery at the same time, the one starting later was omitted from the study. If needed for completion of the form, questions were addressed to the anesthetist. Outcome data were collected by visiting the patients on the wards and from reviewing the medical charts. In order to reduce behavior bias that could occur due to the presence of the researcher, the first five observed operations were excluded from the study.

Based on the literature and WHO recommendations [2] [3], 28 parameters were selected by the researchers as standards for pediatric anesthesia (S1 Table). These 28 parameters were then divided into 17 structure parameters consisting of the equipment and medicines that should be present in the theatre before any surgery starts, and 11 process parameters of actions taken by staff. Structure and process indices were calculated as the proportion of structure and process parameters present respectively. The indicies were presented as means with 95% confidence intervals and compared using t-tests Adverse events occurring before, during and after the surgery were defined based on anesthesia literature from the 1980’s and forward [8] [9] and included hypoxia, bradycardia and tachycardia (S2 Table).

A data collection tool was designed to assess each anesthetic (S1 Appendix). The tool was then tested during three different operations by two researchers at the researchers’ department. The tool was found to be easy to use and assessments of the patients was found to be identical suggesting good inter-rater reliability.

The hospital and the staff were informed about the purpose of the study and written consent was obtained from the anesthetic staff. Oral consent was obtained from the patients’ guardian; the information was standardized and presented both verbally and written in English or Swahili. Ethical approval was obtained from the National Institute for Medical Research in Tanzania (NIMR/HQ/R.8a/Vol. IX/1508).

## Results

General characteristics of the population can be seen in S3 Table.

### Structure index

Nine parameters were always present and one parameter (bag and mask) was not available for any of the patients (S4 Table). The structure index ranged from 70.6% to 94.1% with a mean of 83.5% [95% Confidence Interval (CI) 81,2–85,8] (S1 Fig).

### Process parameters

The process index consisted of 10 parameters (11 if surgery was longer than 60 minutes) (S5 Table). The mean score was 71.1% [95% (CI) 66,8–75,5] with a range from 50% to 90%. (S2 Fig) The process index was lower than the structure index (p<0,001).

### Total quality index

With the structure and process index combined the average was 78.9% [95% (CI) 74,4–80,2] of parameters fulfilled with a low of 66.7% and high of 88.9%.

### Adverse events

In 29 anesthetics 70 adverse events were observed with a median of 2 (mean 2.5) adverse events per patient and a range from 0 to 7 adverse events per patient.

The most common adverse event was hypoxia at extubation in 20 (69%) patients followed by tachycardia at intubation in 13 (45%) of the patients. Nine patients had an episode of severe hypoxia at extubation and a total of 36 events of hypoxia occurred in the 29 patients. In 97% of the episodes some kind of measure was taken. Bradycardia was uncommon, occurring only 2 times and not leading to severe bradycardia although no treatment was given since the bradycardia resolved spontaneously. Tachycardia occurred 32 times but no treatment was given in any of the cases.

### Outcomes

All patients were taken to the recovery unit (mean duration 43 minutes) and all patients were discharged from the recovery unit alive.

## Discussion

We have developed a tool for evaluating the quality of pediatric anesthesia in low-resource settings and tested it in a hospital in Tanzania. The tool defines a number of structure and process standards that are needed for the safe practice of pediatric anesthesia, and detects the occurrence and severity of adverse events during anesthesia. We found that the structures were better than processes of anesthesia and the quality varied between anesthetics. None of the children died in the operating theatre or in the recovery room.

Muhimbili National Hospital is the main referral hospital in the country and also one of the biggest in East Africa. While the level of resources is generally better than in smaller hospitals, we found several unmet needs. Bag and mask were not available in the theaters, which may increase risk in the case of equipment failure or an unexpected event, nor was a functioning suction apparatus present in all theatres. Increasing such important but low-cost resources for anesthesia, or ensuring their presence in theatres through the use of routines such as checklists may be beneficial. This study was from a single-centre and was cross-sectional in design, so we cannot be certain the results are generalizable to other hospitals in low-income countries. However, our findings are similar to those from similar studies assessing anesthesia for caesarean sections [10] and emergency and critical care in Tanzania [11].

Hypoxia was the most frequent adverse event occurring during the observations and action was taken, with one exception, in all cases. The actions taken when hypoxia occurred were successful and no patient died. Hypoxia has been found to be the main reason for cardiac arrest in settings with limited resources [5]. Good monitoring of oxygen saturation and avoidance of hypoxia should therefore be a mainstay of anaesthesia practice. Although no action was taken in response to the two episodes of bradycardia, the duration of these episodes was short and not considered a serious threat to the patient. Tachycardia is more difficult to interpret: is it pain, an effect of the atropine given, or one of many other possible causes? Tachycardia might be a less useful sign of an adverse event than bradycardia and hypoxia.

The use of checklists and education can be effective in order to provide safe anesthesia in settings lacking resources such as anesthesiologists [12]. Intraoperative cardiac arrest occurs in a higher frequency in developing countries [13] and can in most anesthesia-related cases be prevented [14]. The quality of pediatric anesthesia care in low resource settings could be improved by the use of tools to assess the structure and process of care and the occurrence of adverse events might be lowered improving patient safety.

## Conclusion

Pediatric anesthesia in low resource settings suffers from deficiencies in the structures and processes of providing good quality care. Improvement efforts may be best focused on improving the consistency and quality of the process of care and a reduction in adverse events rather than the structures available. Use of the assessment tool developed for this research could be useful for systematic quality-improvement efforts and to assess the needs in different settings.

## Supporting information

S1 Table Standards for pediatric anesthesia. (DOCX)

S2 Table Adverse event definitions. (DOCX)

S3 Table General characteristics. (DOCX)

S4 Table Structure parameters. (DOCX)

S5 Table Process parameters. (DOCX)

S1 Datasheet (XLSX)

S1 Appendix (PDF)

S1 Fig Distribution of structure index score. (DOCX)

S2 Fig Distribution of process index score. (DOCX)
