# Supplementary material for: Quality of pediatric anesthesia: A cross-sectional study of a university hospital in a low-income country
Source: PLoS One. 2018 Apr 9;13(4):e0194622. doi: 10.1371/journal.pone.0194622 (PMC5890975; doi:10.1371/journal.pone.0194622)
Supplement: S1 Table — (DOCX) [file pone.0194622.s001.docx]

**Table 1.** **Standards for pediatric anesthesia.**

| **Structure** | | **Process** | |
| --- | --- | --- | --- |
| Available before induction | | Before/during anesthesia | |
| Chart | Oxygen | ID-check/Consent | Allergies? |
| Anesthesia system | Facemask | Airway-assessment | Documented last food intake |
| Oropharyngeal airway | Laryngoscope | SpO_2_ in use | WHO-checklist |
| Backup Ambu®-bag | Endotracheal tube | Temperature check | Chart filled |
| Suction | Tilting surgical table | Anesthetic staff present during surgery | Pain relief given |
| SpO_2_ monitoring | Induction Drug | Taken to recovery unit after surgery |  |
| Muscle relaxant | Atropine |  |  |
| Epinephrine | Pain relief |  |  |
| IV-fluid |  |  |  |
