# Supplementary material for: Quality of pediatric anesthesia: A cross-sectional study of a university hospital in a low-income country
Source: PLoS One. 2018 Apr 9;13(4):e0194622. doi: 10.1371/journal.pone.0194622 (PMC5890975; doi:10.1371/journal.pone.0194622)
Supplement: S2 Table — (DOCX) [file pone.0194622.s002.docx]

**Table 2.** **Adverse Event Definitions.**

| Event | Definition | |
| --- | --- | --- |
|  | Moderate | Severe |
| Hypoxia | SaO_2_ < 90 % | SaO_2_ < 80 % |
| Bradycardia | HR -15 below age limit | HR -30 below age limit |
| Tachycardia | HR +15 above age limit | HR +30 above age limit |
|  |  | (HR = Heart rate) |

Age limits in HR (beats per minute)

| 0-1 month | 100-160 | 1-12 month | 90-150 |
| --- | --- | --- | --- |
| 1-2 years | 85-140 | 2-5 years | 80-130 |
| 5-12 years | 70-120 | >12 years | 95-130 |
