# Supplementary material for: Quality of pediatric anesthesia: A cross-sectional study of a university hospital in a low-income country
Source: PLoS One. 2018 Apr 9;13(4):e0194622. doi: 10.1371/journal.pone.0194622 (PMC5890975; doi:10.1371/journal.pone.0194622)
Supplement: S3 Table — (DOCX) [file pone.0194622.s003.docx]

**Table 3. General characteristics.**

| Sex | n | % |  | Age (years) |  |  |
| --- | --- | --- | --- | --- | --- | --- |
| Male | 15 | 50 |  | Mean 2.4 (Range 1.5 m – 5 y) |  |  |
|  |  |  |  |  |  |  |
| Staff grade | n | % |  |  |  |  |
| Physician | 11 | 37 |  |  |  |  |
| Nurse Anaesthetist | 19 | 63 |  |  |  |  |
|  |  |  |  |  |  |  |
| Surgical Department | n | % |  | ASA*-class | n | % |
| Ear, Nose and Throat | 17 | 57 |  | ASA I | 23 | 77 |
| General Paediatric Surgery | 13 | 43 |  | ASA II | 6 | 20 |
|  |  |  |  | ASA III | 1 | 3 |
| Elective | 26 | 87 |  |  |  |  |
| Acute | 4 | 13 |  | ASA-class unknown | 2 | 7 |

* ASA, American Society of Anesthesiologists physical status classification system
