# Supplementary material for: Quality of pediatric anesthesia: A cross-sectional study of a university hospital in a low-income country
Source: PLoS One. 2018 Apr 9;13(4):e0194622. doi: 10.1371/journal.pone.0194622 (PMC5890975; doi:10.1371/journal.pone.0194622)
Supplement: S4 Table — (DOCX) [file pone.0194622.s004.docx]

**Table 4.** **Structure parameters.**

| Structure parameters (n=30) | n | % |
| --- | --- | --- |
| Chart | 30 | 100 |
| Oxygen | 30 | 100 |
| Anesthetic system | 29 | 97 |
| Mask* | 30 | 100 |
| Oropharyngeal airway* | 30 | 100 |
| Laryngoscope and blade* | 18 | 60 |
| Bag and mask | 0 | 0 |
| Endotracheal tube* | 27 | 90 |
| Suction apparatus | 8 | 27 |
| Table with tilting function | 30 | 100 |
| Oxygen saturation monitoring | 29 | 97 |
| Induction Drug | 28** | 97 |
| Relaxation drug | 30 | 100 |
| Atropine | 30 | 100 |
| Epinephrine | 30 | 100 |
| Pain relief | 16 | 53 |
| Intravenous Fluid | 30 | 100 |

* Of suitable size

**n=29
