# Supplementary material for: Quality of pediatric anesthesia: A cross-sectional study of a university hospital in a low-income country
Source: PLoS One. 2018 Apr 9;13(4):e0194622. doi: 10.1371/journal.pone.0194622 (PMC5890975; doi:10.1371/journal.pone.0194622)
Supplement: S5 Table — (DOCX) [file pone.0194622.s005.docx]

**Table 5.** **Process parameters.**

| Process index (n=30) | n | % |
| --- | --- | --- |
| ID and consent for surgery | 30 | 100 |
| Asked about allergies | 26 | 90 |
| Difficult airway evaluation | 8* | 29 |
| Asked about last meal | 12** | 41 |
| Use of saturation monitoring | 25 | 86 |
| WHO Checklist used and presented in theatre | 0 | 0 |
| Temperature checked (surgery >60 minutes) | 0 | 0 |
| Chart filled | 29 | 97 |
| Presence of anesthetist | 29 | 97 |
| Post op pain relief given | 25 | 83 |
| Taken to post op unit | 30 | 100 |

*n=28

**n=29
