# Supplementary material for: Quality of pediatric anesthesia: A cross-sectional study of a university hospital in a low-income country
Source: PLoS One. 2018 Apr 9;13(4):e0194622. doi: 10.1371/journal.pone.0194622 (PMC5890975; doi:10.1371/journal.pone.0194622)
Supplement: S1 Appendix — (PDF) [file pone.0194622.s007.pdf]

Assessment Tool for Pediatric Anesthesia
Developed by Oskar Andersson, Karolinska University Hospital

Researcher's name .... Date... Time taken in to theatre .... Research number...........................

Patient's name... Age............................ Hosp number .... Post-op ward... Staff grade..........................

ASA classification........................ O Known by anesthesist Weight .... Height .... Pre-op Hb........................... O Blood type O Cross-match

Elective or Acute O Elective O Acute Surgery.. O IV present at arrival

Are the following in the operating theatre?

Anaesthetic chart	YES	NO	Comment	Tracheal tube – suitable for weight	YES	NO	Comment	Induction Drug	YES	NO	Comment (Type used)
WHO Checklist (presented in theatre)	YES	NO	Comment	Suction apparatus – functioning	YES	NO	Comment	Muscle relaxation	YES	NO	Comment (Type used)
Oxygen supply	YES	NO	Comment	Bougie – suitable size*	YES	NO	Comment	Atropine	YES	NO	Comment
Bag, mask and breathing system – suitable for weight	YES	NO	Comment	Tilting table	YES	NO	Comment	Adrenaline	YES	NO	Comment
Facemask – suitable size	YES	NO	Comment	ECG	YES	NO	Comment	Intra-operative pain relief	YES	NO	Comment
Oro-pharyngeal airway	YES	NO	Comment	Pulse Oximeter	YES	NO	Comment	IV-fluid	YES	NO	Comment (Type used)
Laryngoscope – w/ functioning light and proper size	YES	NO	Comment	Blood Pressure monitor (with suitable size of cuff)	YES	NO	Comment	Neostigmine*	YES	NO	Comment
Bag and mask (Back up) - suitable for weight	YES	NO	Comment	Neck pillow for infants	YES	NO	Comment	Emergency drugs* (Succinylcholin, Atropine, Adrenaline, Pentothal)	YES	NO	Comment

During the operation, are the following things done?

Beginning of op	Patient's identify, consent and type of procedure confirmed	Done	Not done	Comment
	Patient asked if they have any allergy	Done	Not done	Comment
	Performed a difficult airway evaluation	Done	Not done	Comment
	Patient's last time for food intake known	Done	Not done	Comment
	Pulse oximeter on patient and functioning	Done	Not done	Comment
	WHO Checklist (Time out)	**Done**	**Not done**	**Comment**
	Method of induction used	IV	Gas	Comment (Type used)

Number of performed intubation trials	1	2	3	4 or more		Number of performed trials for IV	1	2	3	4 or more
Cries at induction	Yes	No								

During the operation, are the following things done?					
Peri-op	Temperature checked if surgery >6o min	Done	Not done	**Not indicated**	Comment
	Intra-operative fluids administrated	Done	Not done	**Not indicated**	Comment
	Blood given (……………………..ml)	Done	Not done	**Not indicated**	Comment

During the operation, did the following events occur?			Check for cause or treatment given?		When did the event occur?			
Hypoxia <90%	YES	NO	Done	Not done	Induction	Peri-op	Extubation	Comment
Severe Hypoxia <80%	YES	NO	Done	Not done	Induction	Peri-op	Extubation	Comment
Bradycardia (-15)	YES	NO	Done	Not done	Induction	Peri-op	Extubation	Comment
Severe Bradycardia (-30)	YES	NO	Done	Not done	Induction	Peri-op	Extubation	Comment
Tachycardia (+15)	YES	NO	Done	Not done	Induction	Peri-op	Extubation	Comment
Severe tachycardia (+30)	YES	NO	Done	Not done	Induction	Peri-op	Extubation	Comment

During the operation, are the following things done?					
End of op	Blood loss evaluated at end of surgery	Done	Not done	Comment	
	Anaesthetic chart filled in	Done	Not done	Comment	
	WHO Checklist Sign Out	Done	Not done	Comment	
	Presence throughout of a trained anaesthetist	Done	Not done	Comment	
	Postoperative pain relief planned or given	Done	Not done	Comment	
	Patient taken to recovery unit	Done	Not done	Comment	
	When was the extubation performed?	Early	First sign of gagging	Patient awake	Comment
	Cries when extubated	Yes	No		

Outcome

Estimated Blood Loss		ml
MAO Score (see below)		p

Re-operation needed ○ Yes ○ No Date of re-op…………………….. Time of re-op……………………..
Time at recovery unit for surveillance ………………………………………….
○ Alive when discharged from hospital Date of discharge…………………….. Time of discharge……………………..
○ Died in hospital Date of death…………………….. Time of death……………………..
Cause of death in notes………

	Age			
	New born	1-11 m	2 y	4 y
Heart rate	80 - 170	90 - 180	85 - 160	80 - 140
SBP	50-75	75 - 100	75-110	80-115
BF	30 - 60	30 - 60	25 - 50	15 - 30

MAO SCORE	
5	**Uneventful** course of anaesthesia, no complications, comfortable patient.
4	**Smooth** anaesthesia, with only **slight discomfort** for patient, surgeon or anaesthetist, not necessitating special therapeutic action.
3	**Eventful** course of anaesthesia, with minor complications causing slight or temporary discomfort for patient, surgeon or anaesthetist, which necessitated minor therapeutic action which did not interfere with the basic (or intended) anaesthetic technique. **No morbidity for patient**.
2	Serious events occurred during anaesthesia, with **temporary complications** for patient, or causing gross interference with surgery or anaesthesia, but nevertheless resulting in a successful intervention, with the patient **ultimately making a good recovery** of anaesthesia.
1	Serious events occurred during anaesthesia, with major complications for patient, surgeon or anaesthetist, which necessitated gross interference with surgery or anaesthesia, resulting in a **unfavourable outcome** like serious injury or morbidity, the need for re-intervention or longer than planned hospitalisation.
0	**Patient died** in Operating Theatre whatever the cause
L	Connotation to be used in case **logistic problems** occurred as eg. rupture of essential drugs or malfunction of essential equipment, insufficient light, etc
