# Supplementary material for: Quality of pediatric anesthesia: A cross-sectional study of a university hospital in a low-income country
Source: PLoS One. 2018 Apr 9;13(4):e0194622. doi: 10.1371/journal.pone.0194622 (PMC5890975; doi:10.1371/journal.pone.0194622)
Supplement: S2 Fig — (DOCX) [file pone.0194622.s009.docx]

**Figure 2.** Distribution of process index score.
